# Induction of Apoptosis and Effect on the FAK/AKT/mTOR Signal Pathway by Evodiamine in Gastric Cancer Cells

**DOI:** 10.3390/cimb44090298

**Published:** 2022-09-19

**Authors:** Ji Yeong Yang, Hyun Jun Woo, Pyeongjae Lee, Sa-Hyun Kim

**Affiliations:** 1 Crop Foundation Research Division, National Institute of Crop Science (NICS), Rural Development Administration (RDA), Wanju 55365, Korea; 2 Department of Clinical Laboratory Science, Semyung University, Jecheon 27136, Korea; 3 School of Industrial Bio-Pharmaceutical Science, Semyung University, Jecheon 27136, Korea; 4 BK21 FOUR KNU Creative BioResearch Group, School of Life Sciences, Kyungpook National University, Daegu 41566, Korea

**Keywords:** apoptosis, evodiamine, gastric cancer cells, STAT3, mTOR

## Abstract

Evodiamine isolated from *Evodia rutaecarpa* has been known to have anti-tumor activity against various cancer cell types. Although there have been reports showing the inhibitory effect of evodiamine on cell survival of gastric cancer cell, it is not clearly explained how evodiamine affects the expression and modification of proteins associated with apoptosis and upstream signal pathways. We confirmed the cytotoxic activity of evodiamine against AGS and MKN45 cells by a WST assay, cell morphological change, and clonogenic assay. The apoptotic cells were evaluated by Annexin V/PI analysis and Western blot and the expressions of apoptosis-related molecules were confirmed by Western blot. Evodiamine promoted apoptosis of AGS gastric cancer cells through both intrinsic and extrinsic signal pathways in a time- and dose-dependent manner. Evodiamine attenuated the expression of anti-apoptotic proteins, including Bcl-2, XIAP, and survivin, and elevated that of the pro-apoptotic protein Bax. Evodiamine also suppressed the FAK/AKT/mTOR signal pathway. Based on these results, we expect that the results from this study will further elucidate our understanding of evodiamine as an anti-cancer drug.

## 1. Introduction

Gastric cancer is the fifth most-common malignancy worldwide, according to the World Health Organization (WHO) in 2018. The republic of Korea has the highest national incidence, with almost 60 per 100,000 new cases annually for males [1]. Among the different types of treatments available for patients with gastric cancer, surgery is mainly used for patients diagnosed at an early stage. Chemotherapy helps to reduce the amount of cancer before surgery or to remove any remaining cancer cells after surgery [2,3].

Evodiamine is a naturally occurring compound extracted from traditional Chinese herbs and is a well-known anti-tumor agent, potentially working against various cancer cell lines [4,5,6,7,8]. Jia Du et al. showed that evodiamine induces apoptosis through the Bcl-2-associated X protein (Bax)/Bcl-2 pathway in MDA-MB-231 human breast cancer cells [4]. Fan Yang et al. evaluated the efficacy of evodiamine against HepG2 hepatocellular carcinoma cells and showed that evodiamine suppressed AKT and increased Bax/Bcl-2 and cleaved-caspase-3 [5]. Li Lin et al. reported that treatment with evodiamine downregulated survivin and Bcl-2 in A549 human lung cancer cells [6]. Human ovarian cancer cells, HO-8910PM, treated with evodiamine led to the activation of caspase-8, caspase-9, and caspase-3 and the cleavage of poly (ADP-ribose) polymerase (PARP) [7]. Wei WT et al. showed that combination therapy with gemcitabine and evodiamine enhanced the anti-tumor efficacy by inhibiting the activities of phosphatidylinositol-3-kinase (PI3K), protein kinase B (AKT), cAMP-dependent protein kinase A (PKA), mammalian target of rapamycin (mTOR), and phosphatase and tensin homolog (PTEN), as well as the activation of nuclear factor kappa-light-chain-enhancer of activated B cells (NF-κB) and expression of NF-κB-regulated products in SW1990 human pancreatic cancer cells [8].

Several reports demonstrated that evodiamine inhibits proliferation and induces apoptosis in gastric cancer cells. Yang L et al., Shen H et al., and Wen Z et al. suggested that evodiamine inhibited the cell growth and induced caspase-dependent apoptosis in SGC7901 gastric cancer cell lines [9,10,11]. Rasul A et al., Yang L et al., and Wen Z et al. reported that evodiamine induced apoptosis by regulating Bax and Bcl-2 expression in SGC7901 cells [9,11,12]. These apoptotic effects of evodiamine on gastric cancer cells have been studied using SGC7901 cell lines, but not the AGS and MKN45 gastric cancer cells. Therefore, in this study, we determined whether the anti-cancer effect of evodiamine exists in AGS and MKN45 cells. Furthermore, more detailed mechanisms of evodiamine inducing apoptosis, including the inhibitor of apoptosis proteins (IAPs), signal transducer and activator of transcription 3 (STAT3), and focal adhesion kinase (FAK)/AKT/mTOR pathway, were examined.

## 2. Materials and Methods

### 2.1. Cell Culture

The human gastric adenocarcinoma cell lines AGS and MKN45 were used in the current study. AGS gastric adenocarcinoma cells (ATCC CRL-1739) were purchased from the Korean Cell Line Bank (Seoul, Korea) and cultured in Dulbecco’s Modified Eagle’s medium (DMEM, BRL Life Technologies, Grand Island, NY, USA) supplemented with 10% fetal bovine serum (FBS, BRL Life Technologies) and streptomycin–penicillin (100 μg/mL and 100 IU/mL) (BRL Life Technologies). MKN45 cells were cultured in Roswell Park Memorial Institute (RPMI) 1640 medium (BRL Life Technologies) supplemented with 10% FBS and streptomycin–penicillin (100 μg/mL and 100 IU/mL). The images were taken by an inverted microscope under 400× magnification.

### 2.2. WST Cell Viability Assay Using EZ-Cytox

AGS cells and MKN45 cells (1 × 10^4^ per well) were seeded in 96-well tissue culture plates. After 24 h, cells were treated with various concentrations (0.5–20 μM) of evodiamine (≥98% purity; Sigma-Aldrich, St. Louis, MO, USA) dissolved in 100% dimethylsulfoxide (DMSO, ≥99.7% purity; Sigma-Aldrich) for 24 h. The cells were then subjected to water soluble tetrazolium salt (WST) assay by using EZ-Cytox cell viability assay kit (Daeil Lab Service, Seoul, Korea) according to manufacturer’s instruction. The absorbance was measured at 450 nm using NanoQuant Infinite M200 (Tecan, Mannedorf, Switzerland).

### 2.3. Clonogenic Assay

The colony-formation assay was performed as described [13]. AGS and MKN45 cells were plated in 24-well plates at a density of 500 cells per well overnight. The following day, cells were treated with evodiamine (0, 1, 5, and 10 μM) and the medium was replaced with fresh medium containing evodiamine every 3 days. After a 9-day treatment period, the medium was removed and covered the cells with 100% methanol at 25 °C. After 20 min, the methanol was removed and rinsed with water. Colonies were stained with crystal violet (0.5% in 25% methanol) for 5 min and then cells were washed with water until excess dye was removed. After overnight to dry the plate, colony numbers were assessed visually and colonies containing >50 normal-appearing cells were counted.

### 2.4. Annexin V and PI Staining

Annexin V and propidium iodide (PI) staining was performed by using Annexin V-FITC Apoptosis Detection Kit I (BD Biosciences, Franklin Lakes, NJ, USA) according to the manufacturer’s instruction. The cultured cells were washed twice with cold PBS, trypsinized, and centrifuged at 3000 rpm for 5 min. The cells were resuspended in binding buffer at a concentration of 5 × 10^5^ cells/mL and transferred 100 μL of cell suspension in a 5 mL test tube. Then 5 μL of Annexin V-FITC and 5 μL of PI were added to the cell suspension and the mixture was incubated for 15 min at room temperature in the dark. After adding 400 μL of binding buffer to each tube, samples were analyzed by flow cytometry.

### 2.5. Protein Extraction and Western Blot

AGS cells or MKN45 cells (4 × 10^5^ per well) were seeded in 6-well plates. After 24 h, cells were treated with the indicated concentrations (0, 1, 5, and 10 μM) of evodiamine or with 10 μM of evodiamine for the indicated time periods (0, 6, 12, and 24 h). After that, the cells were lysed at 4 °C with a radio immunoprecipitation assay (RIPA) lysis buffer (Millipore, Burlington, MA, USA) containing a protease inhibitor cocktail (Sigma-Aldrich). Proteins were quantified by a Lowry Protein assay (Bio-Rad, Hercules, CA, USA). Protein samples were separated by SDS-polyacrylamide gel and the immune-labeled proteins were visualized using enhanced chemiluminescence (ECL, Thermo Fisher Scientific, Waltham, MA, USA).

### 2.6. Statistical Analysis

Data in the bar graphs are presented as the mean ± standard error of the mean (SEM). All the statistical analyses were performed using GraphPad Prism 7.0 software (GraphPad Software, San Diego, CA, USA). All the data were analyzed by unpaired Student’s *t*-tests and a *p* < 0.05 was considered to be statistically significant.

## 3. Results

### 3.1. Evodiamine Reduces Cell Viability of Gastric Cancer Cell Lines

**Figure 1:**
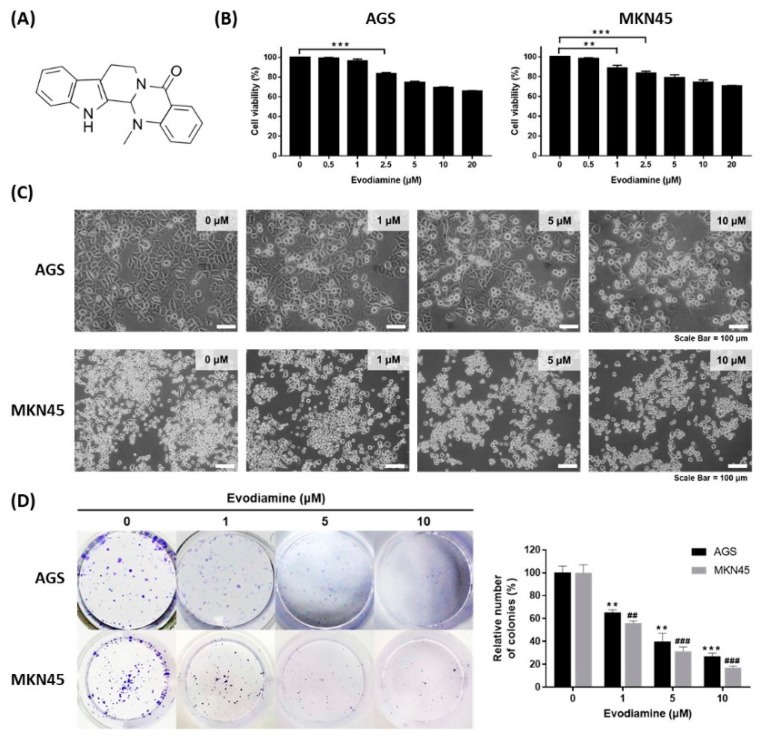
Inhibitory effect of evodiamine on the cell viability of gastric cancer cell lines. (**A**) Chemical structure of evodiamine. (**B**) Gastric cancer cells, AGS and MKN45, were treated with the indicated doses of evodiamine (0, 1, 5, and 10 μM) for 24 h and cell viability was measured by WST assay. Data were from three independent experiments and analyzed by unpaired Student’s *t*-tests (** *p* < 0.01 and *** *p* < 0.001). (**C**) The images were captured using an inverted microscope (×400). (**D**) Clonogenic growth inhibition by evodiamine was evaluated by clonogenic assay and representative images of cells stained with crystal violet are shown. Colonies containing >50 normal-appearing cells were counted visually, and the percentage inhibition of colony formation is illustrated as a graph. Data were from three independent experiments and analyzed by unpaired Student’s *t*-tests. ** *p* < 0.01 and *** *p* < 0.001 vs. the non-treated control in AGS cells; ^##^ *p* < 0.01 and ^###^ *p* < 0.001 vs. the non-treated control in MKN45 cells.

The chemical structure of evodiamine is shown in Figure 1A. The effect of evodiamine on cell viability in gastric cancer cells was evaluated using the human gastric adenocarcinoma cell lines AGS and MKN45. Cells were treated with various concentrations of evodiamine for 24 h and then a WST assay was performed to examine the cell viability. AGS cells treated with 2.5 μM evodiamine showed an approximately 16.7% reduction in viability (Figure 1B). In MKN45 cells, cell viability decreased by 11.3% and 16.3% at 1 μM and 2.5 μM evodiamine, respectively (Figure 1B). In subsequent experiments, evodiamine at a concentration of 1, 5, and 10 μM (which reduced cell viability to approximately 70% at 24 h) was used.

The cytotoxic effects of evodiamine were also confirmed by an inverted microscopic analysis of AGS or MKN45 cells treated with 1, 5, and 10 μM for 24 h. Cells were detached from the culture plates on which they were growing compared to non-treated control cells, which was parallel to the decrease in cell viability (Figure 1C). In addition, evodiamine significantly reduced colony formation in both AGS and MKN45 cells in a dose-dependent manner. Treatment of 10 μM evodiamine for 9 days led to 73% and 83% inhibition in the growth of AGS and MKN45 cells, respectively (Figure 1D). These results suggest that evodiamine effectively inhibits the cell viability of gastric cancer cells.

### 3.2. Evodiamine Induces Apoptosis in AGS and MKN45 Cells

**Figure 2:**
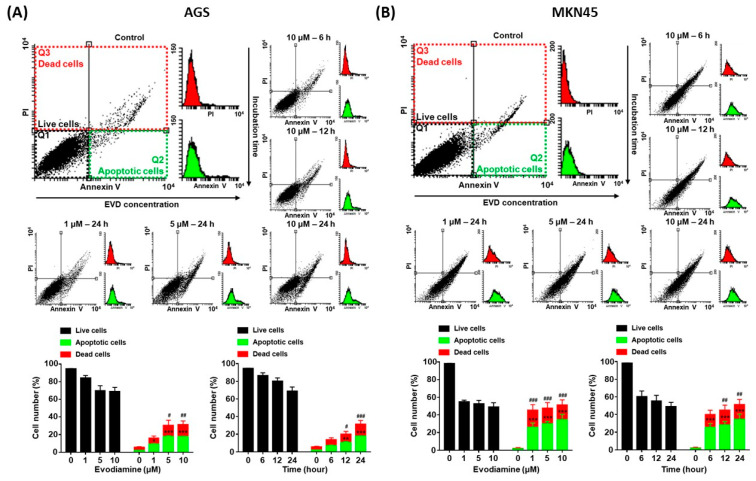
Annexin V and PI staining of AGS and MKN45 cells after evodiamine treatment. (**A**) AGS and (**B**) MKN45 cells were treated with 10 μM evodiamine for 6, 12, or 24 h and 1, 5, or 10 μM evodiamine for 24 h, stained with annexin V-FITC and PI, and subjected to flow cytometry. Stained cells were analyzed and illustrated on the quadrant by CellQuestPro software. Percentage of cells in apoptosis was analyzed and illustrated as a graph. Data are presented as the mean ± SEM. * *p* < 0.05, ** *p* < 0.01, and *** *p* < 0.001 vs. the apoptotic cells control; ^#^ *p* < 0.05, ^##^ *p* < 0.01, and ^###^ *p* < 0.001 vs. the dead cells control. EVD, evodiamine.

Since evodiamine has been reported to induce apoptosis in several types of cancer cell lines [4,5,6,7,8], it was investigated whether decreased cell viability was caused by apoptosis in the current study. AGS and MKN45 cells were treated with 10 μM of evodiamine for 6, 12, or 24 h and 1, 5, or 10 μM of evodiamine for 24 h, and then compared with the evodiamine non-treated control group. As a result of the flow cytometric analysis, apoptotic cells were increased in a dose- and time-dependent manner. After exposure to 10 μM evodiamine for 24 h, in AGS and MKN45 cells, the apoptosis ratio was increased up to 17.6% and 34.6%, respectively (Figure 2).

**Figure 3:**
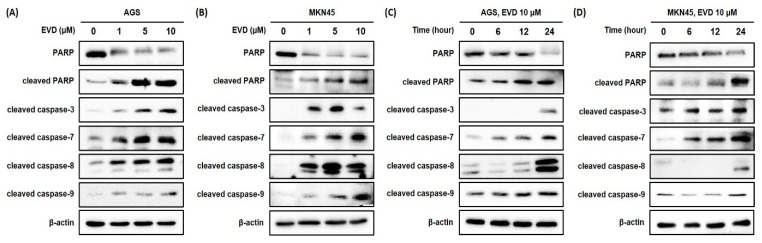
Evodiamine stimulates induction of PARP cleavage and the cleavage of caspase-3 and caspase-7 in AGS and MKN45 cell. Indicated doses of evodiamine (0, 1, 5 and 10 μM) for 24 h were applied to (**A**) AGS and (**B**) MKN45 cells. A total of 10 μM evodiamine for the indicated time periods (0, 6, 12, and 24 h) was applied to (**C**) AGS and (**D**) MKN45 cells. The cell lysates were subjected to Western blot to evaluate the levels of PARP, cleaved PARP, and cleaved caspase-3, -7, -8, and -9. β-actin was used as an internal control. EVD, evodiamine.

For further confirmation of the induction of apoptosis after evodiamine treatment in AGS and MKN45 cells, the cleavage of PARP was assessed by Western blot analysis. It was observed that the full-length form of PARP decreased comparably to the increased cleaved form in a dose- and time-dependent manner (Figure 3). Based on these results, evodiamine treatment significantly induces apoptosis in AGS and MKN45 gastric cancer cells in a dose- and time-dependent manner. Furthermore, these results are in line with previous reports that evodiamine-induced cell death is associated with apoptosis in different cancer cell types.

### 3.3. Evodiamine Reduces Cell Viability via Caspase-Dependent Apoptosis in AGS and MKN45 Cells

Cleavage of PARP is accomplished by effector caspase, caspase-3, and caspase-7, and caspase-8 and caspase-9 are upstream molecules of the effector caspase. To investigate whether evodiamine-induced apoptosis in AGS and MKN45 cells is caspase-dependent, the caspase-3, -7, -8, and -9 activities were determined by Western blot analysis. Evodiamine treatment in AGS and MKN45 cells significantly increased the activities of caspase-3, -7, -8, and -9 in a dose- and time-dependent manner (Figure 3). These results showed that both the initiator caspases (caspase-8 and caspase-9) and effector caspases (caspase-3 and caspase-7) were activated by evodiamine treatment in AGS and MKN45 cells. It is indicated that the activation of classical caspases contributes to apoptotic cell death induced by evodiamine treatment in gastric cancer cells.

### 3.4. Evodiamine Affects the Expressions of Mitochondrial Apoptosis-Related Proteins

**Figure 4:**
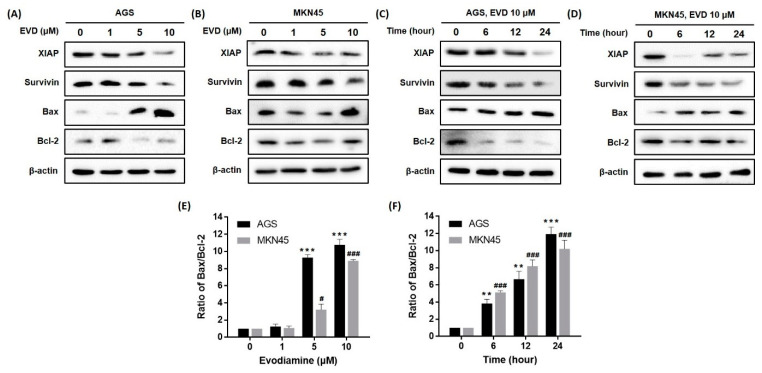
Effect of evodiamine on the expression of the anti- and pro-apoptotic molecules in AGS and MKN45 cells. Indicated doses of evodiamine (0, 1, 5, and 10 μM) for 24 h were applied to (**A**) AGS and (**B**) MKN45 cells. A total of 10 μM evodiamine for the indicated time periods (0, 6, 12, 24 h) was applied to (**C**) AGS and (**D**) MKN45 cells. The cell lysates were subjected to Western blot to evaluate the levels of XIAP, survivin, Bax, and Bcl-2. β-actin was used as an internal control. (**E**,**F**) The intensity of Bax and Bcl-2 was normalized to β-actin and the ratio was calculated. Data are presented as the mean ± SEM of three independent experiments and analyzed by a Student’s *t*-test. ** *p* < 0.01 and *** *p* < 0.001 vs. the non-treated control in AGS cells; ^#^ *p* < 0.05 and ^###^ *p* < 0.001 vs. the non-treated control in MKN45 cells. EVD, evodiamine.

Previous studies have indicated the apoptotic cascade can be inhibited by the inhibitors of apoptosis proteins (IAPs) via directly binding to caspase [14]. Among the IAPs, XIAP inhibits various caspases [14] and survivin inhibits intrinsic pathways through binding to caspase-9 [14]. We confirmed that the protein expressions of XIAP and survivin were reduced by evodiamine treatment in a dose- and time-dependent manner (Figure 4).

The mitochondrial pathway of apoptosis is also dependent upon the Bcl-2 family of proteins, which includes both pro-apoptotic proteins, such as Bcl-2, and anti-apoptotic proteins, such as Bax [15]. As a result, a dose- and time-dependent increase in Bax expression and a decrease in Bcl-2 expression were observed in both AGS and MKN45 cells (Figure 4). In addition, the Bax/Bcl-2 ratio increased in a dose- and time-dependent manner. Especially, the ratio reached an 11.3- and 9.6-fold increment as compared to the non-treated cells following treatment with 10 μM evodiamine for 24 h in AGS and MKN45 cells, respectively (Figure 4E,F).

Collectively, these results indicated that activation of caspase-9 and the subsequent induction of apoptosis by evodiamine can be explained because evodiamine repressed the XIAP, survivin, Bcl-2 levels, and anti-apoptotic proteins, and increased the Bax expression, a pro-apoptotic protein, in AGS and MKN45 gastric cancer cells.

### 3.5. Evodiamine Inhibits FAK/AKT/mTOR Signaling Activation in AGS and MKN45 Cells

The FAK/AKT/mTOR signaling pathway is involved in the regulation of mitochondrial apoptosis, which plays a critical role in cellular growth and apoptosis [16,17]. Therefore, Western blot was applied to detect the protein expressions of components in the pathway, including FAK, phosphorylated-FAK (p-FAK), AKT, phosphorylated-AKT (p-AKT), mTOR, and phosphorylated-mTOR (p-mTOR).

**Figure 5:**
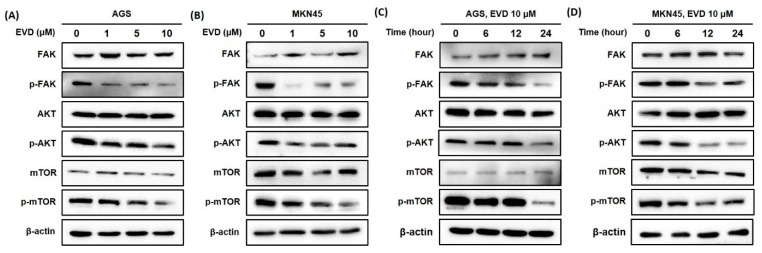
Evodiamine inhibits phosphorylation of FAK, AKT, and mTOR in AGS and MKN45 cells. Indicated doses of evodiamine (0, 1, 5, and 10 μM) for 24 h were applied to (**A**) AGS and (**B**) MKN45 cells. A total of 10 μM evodiamine for the indicated time periods (0, 6, 12, and 24 h) were applied in (**C**) AGS and (**D**) MKN45 cells. The cell lysates were subjected to Western blot to evaluate the levels of FAK, p-FAK, AKT, p-AKT, mTOR, and p-mTOR. β-actin was used as an internal control. EVD, evodiamine.

As shown in Figure 5, the results of the Western blot revealed that the protein expression levels of the phosphorylated form of FAK were increasingly inhibited upon treatment, with increasing exposure times and concentrations of evodiamine, while the levels of FAK proteins were constant despite evodiamine treatment in AGS and MKN45 cells. Furthermore, the protein levels of the phosphorylated form of AKT and mTOR decreased by evodiamine treatment, while the levels of AKT and mTOR proteins were constant despite evodiamine treatment in AGS and MKN45 cells. Collectively, these experimental data revealed a potent inhibitory effect of evodiamine on FAK/AKT/mTOR signaling, leading to apoptosis in human gastric cells treated with evodiamine.

### 3.6. Evodiamine Inhibits the Activation of STAT3 Signaling in AGS and MKN45 Cells

Bcl-2 family proteins are known to be regulated by the activation of STAT3, those involved in controlling the fundamental cellular processes of apoptosis, including c-Myc [18]. To determine whether the change in mitochondrial apoptosis-related proteins is induced by inhibition of STAT3 activation, Western blot was performed to detect the STAT3 and phospho-STAT3 (p-STAT3) protein with the transcription factor c-Myc.

**Figure 6:**
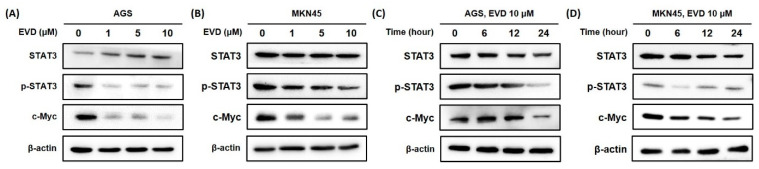
Evodiamine inhibits STAT3 activation and cMyc expression in AGS and MKN45 cells. Indicated dose of evodiamine (0, 1, 5, and 10 μM) for 24 h were applied to (**A**) AGS and (**B**) MKN45 cells. A total of 10 μM evodiamine for the indicated time periods (0, 6, 12, 24 h) were treated in (**C**) AGS and (**D**) MKN45 cells. The cell lysates were subjected to Western blot to evaluate the levels of STAT3, p-STAT3, and c-Myc. β-actin was used as an internal control. EVD, evodiamine.

It was confirmed that the phospho-STAT3 protein was decreased in a dose- and time-dependent manner when evodiamine was applied to AGS and MKN45 cells. The expression of cMyc was also diminished by evodiamine treatment (Figure 6). The result suggested that evodiamine suppressed the activation of STAT3, which downregulated the expression of c-Myc. This can lead to a decrease anti-apoptotic proteins, such as Bcl-2, which consequently seemed to result in apoptosis of gastric cancer cells.

## 4. Discussion

It was shown that the cytotoxic activity of evodiamine against AGS and MKN45 cells, exhibited by the WST assay and cell morphological change (Figure 1B,C), led to inhibiting colony formation (Figure 1D). This is the first report concerning the effect of evodiamine against MKN45 cells. AGS cells were inhibited by 16.7% at a 2 μM concentration of evodiamine for 24 h (*p* < 0.001), as shown in Figure 1B, which is a similar concentration to previous studies [11,19]. It supports that evodiamine has a significant anti-cancer effect on gastric cancer in addition to the previously reported effects against a different line of gastric cancer cell, SGC7901 [9,10,11,12,20]. Although the cytotoxicity of evodiamine against normal cells was not confirmed in this study, Lee T J et al. reported that evodiamine had cytotoxic activity against human leukemic U937 cancer cells but not against normal peripheral blood mononuclear cells [21].

Exposing cells to cytotoxic compounds can result in several cell fates. This study focused on evodiamine-induced apoptosis. The results of Annexin V/PI analysis and Western blot detected cleaved PARP, showing that evodiamine induced apoptotic cell death in a dose- and time-dependent manner in AGS and MKN45 cells (Figure 2 and Figure 3). Although apoptosis is stimulated by numerous external or internal signal stimuli, it is generally considered that the common pathway of apoptosis is the activation of the caspase family [22,23]. In the current study, evodiamine-induced apoptosis was accompanied by activation of caspase-3, -7, -8, and -9 (Figure 3). It means that apoptosis induced by evodiamine in gastric cancer cells was mediated by both intrinsic and extrinsic cascades, which is consistent with Li Yang et al.’s report.

Among the IAPs, XIAP typically inhibits caspase-3, -7, and -9, and survivin inhibits the intrinsic pathway through binding to caspase-9 [14]; that is, both XIAP and survivin inhibit caspase-9, which is an initiator caspase of the intrinsic apoptotic cascade. Caspase-9 was activated by evodiamine treatment; therefore, XIAP and survivin expressions were investigated in this study (Figure 4). Bcl-2 family proteins inhibit or activate most of the intrinsic pathways. Especially, the ratio of Bax/Bcl-2 is important to regulate the release of cytochrome *c* from the mitochondria, and is currently being explored as a marker for tumorigenesis and potential targets for treatment [24]. As shown in Figure 4, the ratio of Bax/Bcl-2 was significantly increased after evodiamine treatment. Consequently, evodiamine seems to increase the pore size of the mitochondrial membrane, large enough to allow cytochrome *c* to be released from the mitochondria, which results in activation of the intrinsic pathway in gastric cancer cells.

The FAK/AKT/mTOR signaling pathway is frequently hyperactivated in various cancers, including gastric cancer. As shown in Figure 5, it suggests that evodiamine can act as an AKT/mTOR pathway inhibitor to control apoptosis cell death in evodiamine-treated human gastric cancer cells. Previous reports demonstrated that this pathway is involved in the regulation of mitochondrial apoptosis by regulating the expressions of IAPs [16,17,25,26]. It is reasonable to suggest that downregulation of phosphorylated FAK, AKT, and mTOR expressions by evodiamine treatment leads to the inhibitory effects of evodiamine on intrinsic apoptotic signaling and eventually enhancing apoptosis. Interfering with the FAK function has been a strategy for development of anti-cancer drugs [27,28]. The therapeutic strategies for directly or indirectly blocking the mTOR protein are also attractive and has led to the development of new drugs such as temsirolimus, everolimus, and deforolimus [29,30,31].

The IAP family proteins as well as Bcl-2 family proteins are known to be regulated by the activation of STAT3 [18]. STAT3 proteins are transcriptional activators and upregulate several growth-promoting genes, such as c-Myc, which upregulates anti-apoptotic genes, such as Bcl-2, XIAP, and survivin, and also downregulates pro-apoptotic genes, such as Bax [18,32,33,34]. In this study, evodiamine prevented the phosphorylation of STAT3 and downregulated the c-Myc expression in gastric cancer cells (Figure 6). It may lead to suppression of the Bcl-2 and activation of Bax expression, which eventually induces apoptosis. Furthermore, Barre et al. reported that the transcriptional activity of STAT3 was inhibited by the AKT signaling pathway in T98G cells [35]. mTOR has been studied to phosphorylate STAT3 at serine 727, which is required for the maximal transcriptional activity of STAT3 [36]. Therefore, inhibition of the AKT/mTOR pathway by evodiamine may be further inhibition of STAT3 activation by evodiamine, leading to a further amplification of apoptotic signaling.

Several in vivo studies were performed for toxicity assessment of evodiamine. Liao et al. demonstrated that the toxicity of evodiamine was not observed in a xenograft model and body weight was not affected [37]. On the other hand, evodiamine had a 10% lethal concentration of 354 ng/mL (1.67 μM) and induced cardiac malfunction on zebrafish in vivo [38]. However, lots of anti-tumor drugs are known to cause cardiotoxicity during the treatment period [39]. Considering that the dried unripe fruits of *Evodia rutaecarpa*, the source of evodiamine, have been extensively used for treatment in China [40,41], evodiamine will be potentially applicable for treatment of gastric cancer although it is necessary to pay attention to the prescribed dosage. Another defect of evodiamine is poor solubility in an aqueous medium, which, in turn, considerably attenuates its biological activity in vivo [42]. In order to improve or complement these limitations, it is considered necessary to perform various studies, such as modification of the structure or trapping evodiamine in nanoparticles targeting gastric cancer cells.

According to the WHO in 2018, the fifth most common malignancy worldwide is gastric cancer; it is also the third leading cause of cancer-related morbidity [43]. In particular, more than 50% of gastric cancer cases have occurred in Eastern Asia [44]. Therefore, discovery of possible medications for gastric cancer seems to be necessary, especially for countries of Eastern Asia as well as worldwide. As described in this study and several other studies, it is expected that evodiamine contributes to the improvement or prevention of cancer treatment. However, further studies should aim to evaluate the effects of evodiamine in vivo and to further develop its use as an anti-cancer drug. In addition, it should be undertaken to investigate combination therapy with current chemotherapeutic drugs of gastric cancer and evodiamine, which will increase the translation relevance for considering evodiamine as an anti-cancer agent.

## 5. Conclusions

In the current study, the inhibitory effect of evodiamine on gastric cancer cells and its inhibitory mechanism were investigated. Here, it was found that evodiamine effectively decreased the viability of gastric cancer cells. Several apoptotic pathways appeared to be responsible for induction of apoptosis by evodiamine in gastric cancer cells. Decreased cell viability of gastric cancer cells by evodiamine treatment was mediated by activation of caspase-3, caspase-7, caspase-8, and caspase-9; cleavage of PARP; and subsequent induction of apoptosis. The inhibitors of apoptosis proteins, XIAP and survivin, were decreased by evodiamine, which might be involved in the activation of caspases after evodiamine treatment in gastric cancer cells. Furthermore, evodiamine raised the ratio of Bax/Bcl-2 in gastric cancer cells, which might lead to activate caspase-9, consequently inducing apoptosis. Evodiamine inhibited the phosphorylation of FAK, AKT, and mTOR protein, which seemed to contribute to the increase in Bax and decrease in IAPs. Evodiamine also inhibited STAT3 activation with the level of c-Myc, which seemed to lead to downregulation of the Bcl-2 and IAPs expressions. Collectively, evodiamine inhibits growth of gastric cancer cells by inducing apoptosis, which might be mediated by activation of caspases and inhibition of mitochondrial apoptosis-related proteins, the FAK/AKT/mTOR pathway, and STAT3 signaling.

## Data Availability

The data that support the findings of this study are available from the corresponding author upon reasonable request.
